# Targeting Polyphosphate Kinases in the Fight against Pseudomonas aeruginosa

**DOI:** 10.1128/mBio.01477-21

**Published:** 2021-08-03

**Authors:** Kanchi Baijal, Michael Downey

**Affiliations:** a Department of Cellular and Molecular Medicine, University of Ottawa, Ottawa, Ontario, Canada; b Ottawa Institute of Systems Biology, University of Ottawa, Ottawa, Ontario, Canada

**Keywords:** PPK1, PPK2, *Pseudomonas*, enzyme inhibition, polyphosphate, polyP, gallein

## Abstract

Polyphosphate (polyP) is a universally conserved molecule that plays critical roles in managing bacterial stress responses, in addition to biofilm formation and virulence. The enzymes that make polyphosphate molecules are called polyphosphate kinases (PPKs). Since these enzymes are not conserved in higher eukaryotes, PPKs make excellent therapeutic targets. In a recent paper in *mBio*, Neville and colleagues described gallein, a commercially available G-protein antagonist, as a novel dual-specificity inhibitor against two families of PPK enzymes in Pseudomonas aeruginosa. In this commentary, we discuss the impact of this discovery, outline potential challenges of implementing gallein use in the clinic, and describe how gallein will serve as a fantastic new tool to further fundamental PPK and polyP research in bacteria.

## COMMENTARY

Inorganic phosphates can be joined together in long chains up to thousands of residues in length to form molecules called polyphosphates (also referred to as polyP). While polyP chains are ubiquitous across biological kingdoms, much of the foundational work characterizing their function and regulation was carried out by the late Nobel laureate Arthur Kornberg using bacterial models, such as Escherichia coli and Pseudomonas aeruginosa (1). These bacteria have low levels of polyP under standard growth conditions but rapidly accumulate polyP in response to a wide array of stresses, including amino acid starvation or oxidative stress generated by household bleach (2–4). The enzymes that make bacterial polyP, called polyphosphate kinases (PPKs), hydrolyze the terminal phosphate of ATP or GTP before adding it to growing chains (4). There are multiple unrelated families of PPK enzymes (PPK1 and PPK2), with some PPKs functioning primarily in the generation of phosphorylated nucleotides in addition to potential roles in polyP synthesis (4). Kornberg and others demonstrated that PPK enzymes have roles in virulence, motility, biofilm formation, and the response to cellular stress across diverse species of bacteria (4). Although care must be taken to avoid attributing Δ*ppk* phenotypes solely to changes in polyP metabolism, there are some cases where polyP has been shown to act directly. For example, polyP synthesized in response to cellular stress in E. coli binds to the Lon protease to promote its activity toward free ribosomal proteins and proteins required for DNA replication (5, 6). Moreover, the impact of polyP may extend beyond the bacterial cell itself. Exciting work by Roewe et al. suggests that polyP released from pathogenic E. coli during infection reprograms the immune response of host macrophages (7).

Interestingly, there is no PPK homolog in higher eukaryotes, and the mechanism of polyP synthesis in humans remains largely unknown (8). While this gap in knowledge remains a thorn in the side of polyP researchers dedicated to deciphering roles of human polyP (e.g., blood clotting [9] and neuronal signaling [10]), it highlights an incredible opportunity. Small-molecule inhibitors of PPK enzymes would be expected to decrease bacterial polyP production, along with virulence, biofilm formation, and their ability to resist the stress of antibiotic treatment—all while leaving host cells unaffected. Thus, PPKs are ideal targets for the development of new antibacterial drugs.

Previous efforts in this area have shown promise. An array of *in silico* molecular modeling and candidate-based approaches have been used to identify a few PPK inhibitors, some with 50% inhibitory concentration (IC_50_) values in the lower micromolar (10 μM) range (see a review by Bowlin and Gray [11] for an excellent summary of these). Importantly, many recently described inhibitors show phenotypes that we would expect based on our knowledge of PPK activities, mirroring phenotypes of Δ*ppk* bacteria. Of particular interest is the drug mesalamine. While exhibiting modest inhibition of E. coli PPK *in vitro*, mesalamine treatment at 100 μM decreased stress-induced polyP accumulation by ∼2-fold in uropathogenic E. coli, Vibrio cholerae, and P. aeruginosa (12). What makes mesalamine particularly interesting it that it is already widely prescribed to treat inflammatory bowel diseases such as ulcerative colitis (13). Repurposing approved drugs for new treatments can save precious time and resources in drug design. In their recent article in *mBio* (14), Neville et al. described another small molecule that could be repurposed for inhibition of PPK enzymes from P. aeruginosa.

P. aeruginosa is an opportunistic multidrug-resistant bacterium that causes blood and lung infection as well as sepsis (15). It is a major risk for cystic fibrosis and immunodeficient HIV patients, and its treatment remains a top priority for the World Health Organization (15, 16). P. aeruginosa has stacked the deck in the fight against PPK enzymes, encoding three PPK2 homologs (PPK2A to PPK2C) in addition to PPK1. In Δ*ppk1* mutants, PPK2A and PPK2B can pick up the slack, making enough polyP to allow the cells to proactively exit the cell cycle in response to stressors such as nutrient starvation (17). While this is beneficial for the bacteria, it is bad news for the hosts.

Neville and colleagues fight back with a clever screening strategy based on earlier work showing that extracts from the fruit of Terminalia chebula, rich in ellagic acid derivatives, can inhibit PPK1 (18). They started by screening a small chemical library that included compounds related to ellagic acid, before synthesizing and testing analogs of top performers. The leading compound to emerge from this analysis, with an IC_50_ of 17 μM against PPK1, was identical to a molecule called gallein (14). Gallein is a commercially available fluorescent compound that was once used in histology staining (19) and has more recently gained attention for its ability to inhibit G-protein beta-gamma subunits in mammalian cells (20).

Fortuitously, Neville et al. discovered that in addition to PPK1, gallein has inhibitory activity against all three PPK2 proteins expressed by P. aeruginosa. Thus, gallein is actually a dual-specificity inhibitor, which makes it potentially valuable from a therapeutic standpoint. Indeed, gallein treatment of P. aeruginosa attenuated polyP accumulation, biofilm formation, and bacterial motility (14). Additionally, consistent with the antivirulence activity expected for PPK inhibition, P. aeruginosa infection of Caenorhabditis elegans was reduced after gallein treatment, with no apparent effect on the host (14). In an exciting development, Neville et al. also discovered that cells lacking PPK enzymes or treated with gallein have decreased levels of secreted siderophores called pyoverdine and pyocyanin, which give P. aeruginosa cultures their distinctive green color (15, 21). Pyoverdine chelates iron from host cells and facilitates its uptake by the bacteria, while pyocyanin causes oxidative stress in host cells (15). This is the first description of PPK involvement in the production of these molecules, and the mechanism at play remains unclear. The authors propose an indirect model where cells lacking the polyP required to sequester excess iron proactively downregulate siderophore production as a means of self-protection (14). In contrast, it is tempting to speculate that polyP may directly bind to and activate enzymes that synthesize siderophores or export them from the cell, analogous to what has been described for the Lon protease (6). These enzymes could also be targets for lysine polyphosphorylation, a nonenzymatic posttranslational modification that involves the covalent attachment of long polyP chains to lysine residues of target proteins (22). Of course, defects in siderophore production could also be due to the disruption of PPK activities distinct from polyP metabolism altogether. Nevertheless, the involvement of PPKs in siderophore production remains an exciting area for future research.

A critical question is whether gallein is acting through PPK enzymes. The answer appears to be yes. Specifically, the authors find that gallein has no further impact on polyP accumulation or the other phenotypes described for a mutant strain of P. aeruginosa lacking all four PPK enzymes (14). This classical epistasis experiment suggests PPK enzymes and gallein exert their effects via a common pathway. Since PPK enzymes are particularly important for stress responses, Neville et al. suggest that gallein could be employed to enhance the efficacy of antibiotic treatments that would otherwise have little effect on their own. While gallein is not quite ready for the clinic, there are several critical next steps that the authors can take to move closer toward that goal. First, since only a single lab strain was used in the study, it will be critical to evaluate the sensitivity of gallein against clinically derived isolates. Second, it will be important to understand if (and how) bacteria develop resistance to gallein treatment. Potential resistance mechanisms could include up- or downregulation of polyP-regulated pathways or the activation of drug efflux mechanisms (23). Finally, it will be essential to thoroughly test the clearance of P. aeruginosa in mammalian models of infection (24).

So, is gallein safe for use in humans? The authors show that 100 μM treatment is not cytotoxic in cultured human HEK293T cells (14). However, as a G-protein antagonist, gallein can act on molecular pathways involved in cardiomyocyte signaling, cancer biology, and kidney metabolism (25–27). As such, at least in its current form, gallein use in humans should be approached with care. It is possible that additional analogs of gallein could be generated using medicinal chemistry approaches to improve specificity toward PPKs, while limiting off-target effects. A second critical question is whether gallein can act to inhibit PPK1 and PPK2 enzymes of other bacteria. While this could broaden the utility of gallein as a treatment, it could also result in inhibition of beneficial bacteria that are part of our natural microbiota, leading to dysbiosis (28).

Beyond its potential clinical use in the treatment of bacterial infections, gallein can serve as an excellent reagent to study fundamental aspects of PPK biology in P. aeruginosa, and perhaps other species. By using gallein to shut down the activity of PPK enzymes quickly, researchers can begin to study the contribution of these enzymes and polyP to bacterial physiology before the cells have a chance to adapt to their absence. This approach may help researchers to separate the direct versus indirect effects of PPK enzymes, which remains a critical open question in the field. Moreover, rapid PPK inhibition will allow polyP aficionados to better study the *in vivo* kinetics of polyP degradation by the exopolyphosphatase PPX that degrades chains starting at the end of the molecule (4). Finally, gallein could be exploited in the search for new phenotypes of PPK-deficient cells. Notably, researchers could screen available mutant collections in the presence and absence of gallein to define synthetic lethal relationships and thereby map the landscape of PPK and polyP biology. In this way, beyond a first step toward the clinic, gallein represents an exciting new tool that can be applied to advance research in P. aeruginosa.
